# Cardiac valve calcification is associated with mortality in hemodialysis patients: a retrospective cohort study

**DOI:** 10.1186/s12882-022-02670-5

**Published:** 2022-01-22

**Authors:** Jiuxu Bai, Xiaoling Zhang, Aihong Zhang, Yanping Zhang, Kaiming Ren, Zhuo Ren, Chen Zhao, Qian Wang, Ning Cao

**Affiliations:** 1 Department of Blood Purification, General Hospital of Northern Theater Command, 83 Wen Hua Road, Shenyang, 110016 Liaoning China; 2 Department of Nephrology, Jin Qiu Hospital of Liaoning Province (Geriatric Hospital of Liaoning Province), Shenyang, China

**Keywords:** Cardiac valve calcification, Mortality, Hemodialysis

## Abstract

**Background:**

Cardiac valve calcification (CVC) is common in end-stage renal disease (ESRD). We investigated the effect of CVC on all-cause and cardiovascular (CV) mortality in maintenance hemodialysis (MHD) patients.

**Methods:**

A retrospective cohort study was conducted on 434 hemodialysis patients who underwent echocardiography for qualitative assessment of valve calcification with complete follow-up data from January 1, 2014, to April 30, 2021. The baseline data between the CVC and non-CVC groups were compared. The Kaplan–Meier method was used to analyse all-cause and cardiovascular mortality. The association of CVC with all-cause and cardiovascular mortality was evaluated using multivariate Cox regression analysis.

**Results:**

Overall, 27.2% of patients had mitral valve calcification (MVC), and 31.8% had aortic valve calcification (AVC) on echocardiography. Patients with CVC showed significantly higher all-cause (log-rank *P* < 0.001) and cardiovascular (log-rank *P* < 0.001) mortality rates than patients without CVC. In multivariate regression analyses, MVC (HR: 1.517, *P* = 0.010) and AVC (HR: 1.433, *P* = 0.028) were significant factors associated with all-cause mortality. MVC (HR: 2.340, *P* < 0.001) and AVC (HR: 2.410, *P* < 0.001) were also significant factors associated with cardiovascular mortality.

**Conclusions:**

MVC and AVC increased the risk of all-cause and cardiovascular mortality in MHD patients. Regular follow-up with echocardiography could be a useful method for risk stratification in MHD patients.

## Background

End-stage renal disease (ESRD) substantially increases the risk of cardiovascular morbidity and mortality [1]. Cardiovascular disease (CVD) is the leading cause of death in maintenance dialysis (MHD) patients. Cardiovascular (CV) mortality risk in patients receiving haemodialysis or peritoneal dialysis is observed to be 10 to 20 times that of the general population [2]. Cardiac valve calcification (CVC) plays an important role in cardiac structural changes and the progression to CVD and is frequently seen in patients undergoing dialysis. CVC, including aortic and mitral valve calcification (AVC and MVC), has been reported to occur with a higher prevalence (57.5%) among incident hemodialysis patients [3]. Thus, derangements in calcium-phosphorous metabolism may contribute to CVC.

The association of cardiac valve (mitral and aortic) calcification and clinical outcomes has been studied in patients with end-stage renal disease (ESRD). CVC is correlated with higher cardiovascular and all-cause mortality risk in dialysis patients [4]. To better understand the relationship between valvular calcification and all-cause and CV mortality in MHD patients, we conducted a retrospective cohort study. In this study, we aimed to investigate the association of cardiac parameters, valvular calcification, serum biochemical parameters, and clinical data with risk of cardiovascular or all-cause mortality in MHD patients.

## Methods

This was a retrospective cohort study on 434 MHD patients who received hemodialysis at the General Hospital of Northern Theater Command and were enrolled in the cohort. This study collected data from January 1, 2014, through December 31, 2014, and follow-up data were collected until April 30, 2021. The patients met the following inclusion criteria: (1) age over 18 years; (2) use of hemodialysis for ≥6 months; (3) patients received hemodialysis 3 times per week in 4-h sessions using a dialysate calcium concentration of 1.5 mmol/L; and (4) each patient had complete follow-up data, including echocardiographic parameters. The exclusion criteria were as follows: (1) patients who underwent kidney transplant or were changed to peritoneal dialysis; and (2) primary dilated cardiomyopathy. This study was approved by the Ethics Committee of General Hospital of Northern Theater Command.

All clinical data of MHD patients were collected and consisted of age, dialysis vintage, sex, primary disease, complications, medical history, height, weight, and smoking status. Phosphorus binder medication included calcium-based phosphate binders (calcium carbonate and calcium acetate) and calcium-free phosphate binders (lanthanum and sevelamer). Vitamin D medication included calcitriol and alfacalcidol. Nutritional vitamin D was not included in the study. Vitamin D, including calcitriol or alfacalcidol, was started at a low dose, independent of the initial PTH concentration, and then titrated based on the PTH response. Vitamin D was stopped when calcium>2.5 mmol/L. The intact PTH (iPTH) level was maintained in the range of approximately 2–9 times the upper normal limit for the assay based on the Kidney Disease Improving Global Outcomes (KDIGO) guidelines [5, 6]. The body mass index (BMI) was measured as kilograms divided by the square of height in metres. Blood data were analysed from fasting blood samples obtained from a vein between 7:00 AM and 8:00 AM on the dialysis day within 3 months of the date of ultrasonic echocardiography. Blood tests were performed in the same laboratory using standard laboratory procedures and included haemoglobin (HGB), serum phosphate (P), serum calcium (Ca), albumin (ALB), β2-microglobulin, uric acid, iPTH and 25-hydroxy vitamin D (25(OH)D).

All echocardiographic examinations were performed at our hospital by experienced cardiologists on a nondialysis day. CVC was assessed by colour Doppler echocardiography, and other heart data were obtained. Valve calcification was defined as the presence of bright echoes > 1 mm on one or more cusps of the aortic valve, mitral valve, or mitral annulus [7]. The LV ejection fraction (LV-EF) was measured based on changes in the LV diameter or two-dimensional area between systole and diastole [8]. The left atrial dimension (LAD), left ventricular end-diastolic internal dimension (LVDd), left ventricular posterior wall thickness (LVPWT), and interventricular septal wall thickness (IVST) were obtained from M-mode tracings. The left ventricular mass index (LVMI) was also assessed. The left ventricular mass (LVM) was calculated using the following formula [echocardiographic assessment of left ventricular hypertrophy (LVH): comparison to necropsy findings]: LVM = (1.04 × (IVST + LVDd + LVPWT))^3^ — LVDd ^3^) × 0.8 + 0.6 [9]. The LVMI was obtained by calculating the ratio of the LVM to the body surface area (BSA). The BSA formula for Chinese individuals is S = 0.0061 × height + 0.0124 × W -0.0099 (S, BSA, m2; H, height, cm; W, weight, kg) [10]. LVH was defined as LVMI > 115 g/m^2^ (men) and > 95 g/m^2^ (women) [11].

The endpoint events were all-cause death and cardiovascular death. Cardiovascular death was defined as myocardial infarction, heart failure, arrhythmia, sudden death, stroke, and other CV causes of death. Data for end points were obtained from hospital charts and through telephone interviews with patients conducted by trained reviewers who were blinded to the ultrasonic echocardiography analysis.

Data are expressed as the mean ± SD or frequency (as percentage). Univariate survival analysis was performed with Kaplan–Meier analysis, and the overall significance was calculated by the log-rank test. Comparisons between groups were performed by an unpaired t-test or the nonparametric Wilcoxon rank-sum test in the case of nonnormally distributed variables. Continuous variables were compared between the groups using Student’s t test or the Mann-Whitney U-test, as appropriate. Categorical data were compared between the groups by the chi-square test. The Cox proportional hazards model was used to estimate the relative risk of all-cause mortality and cardiovascular mortality for different variables. All baseline variables with *P* < 0.05 by univariate analysis were entered into a multivariate model to identify independent predictors for the end point. All statistical tests were performed at a two-sided significance level of 0.05. Statistical Package for the Social Sciences (SPSS for Windows, IBM Corp, USA) version 22.0 was used for data analysis.

## Results

**Fig. 1:**
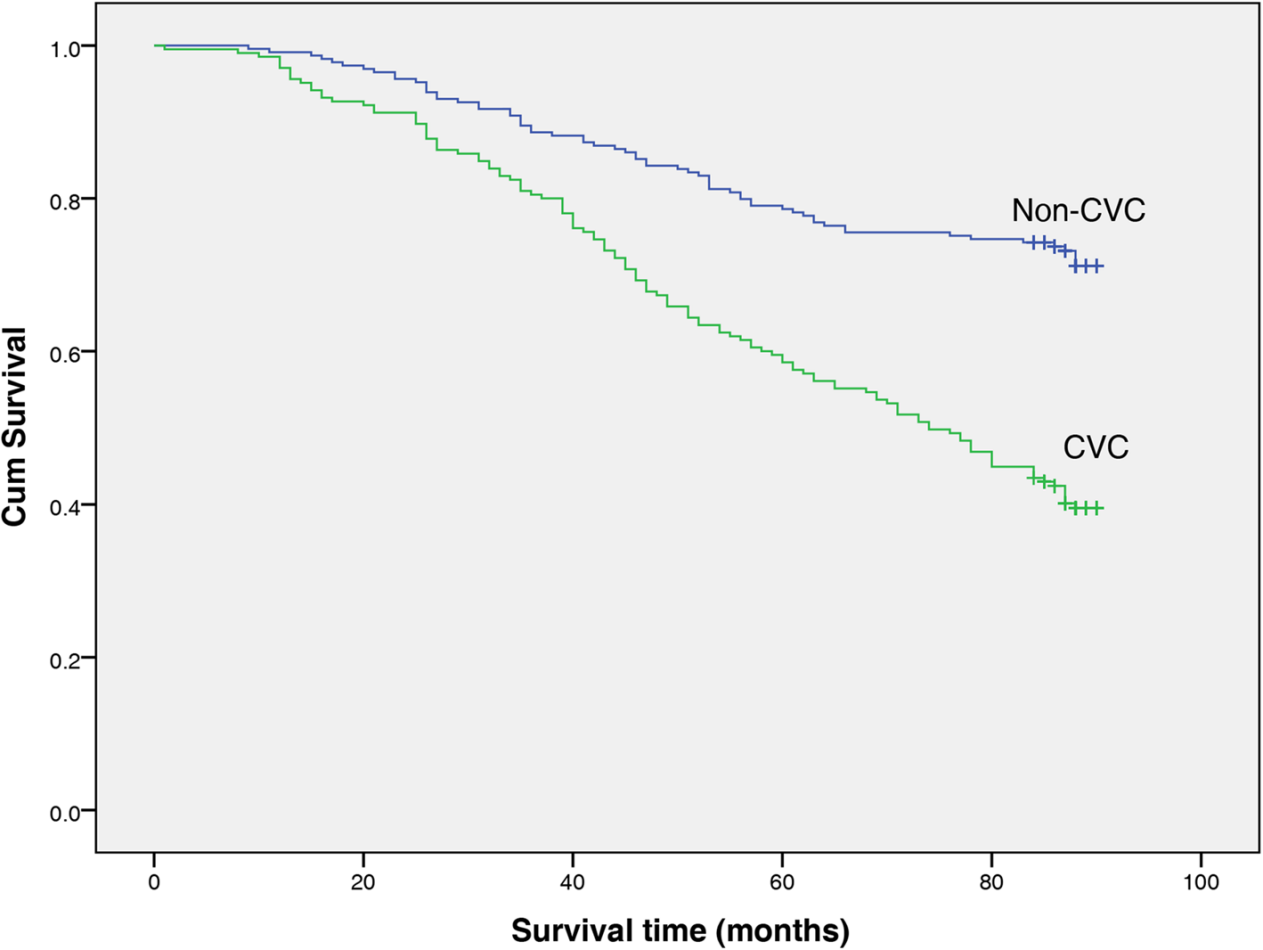
Kaplan–Meier graph of all-cause mortality risk in the non-CVC and CVC groups

**Fig. 2:**
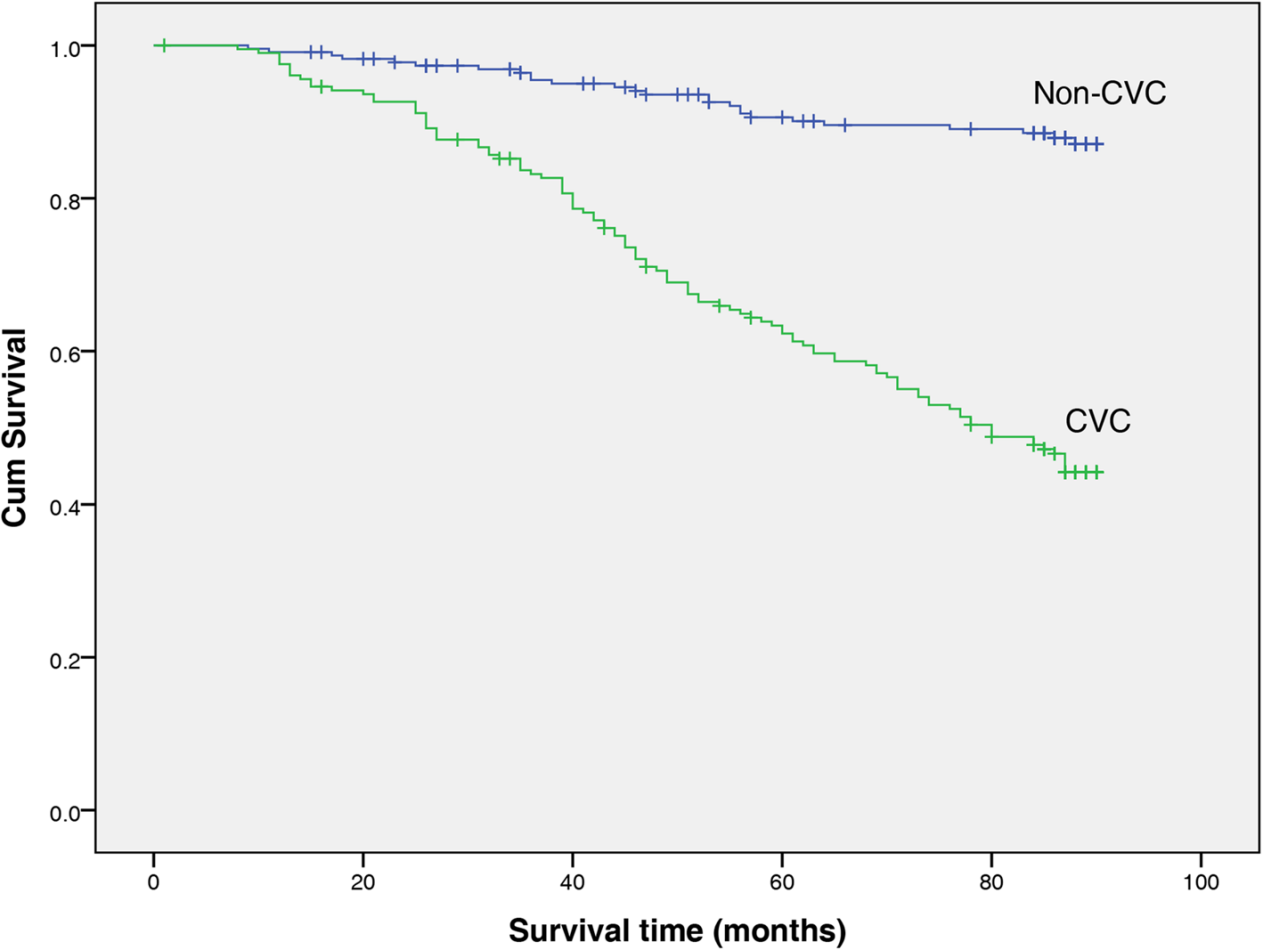
Kaplan–Meier graph of cardiovascular mortality risk in the non-CVC and CVC groups

During a mean follow-up period of 70 months, 187 (43.1% of total) patients died (135 of these from CVD causes). Kaplan–Meier analyses were performed to examine the univariate association between the presence of CVC and outcome. Figures 1 and 2 show the relationship between CVC and death from all causes and CV mortality. The all-cause mortality rate was significantly higher among patients with CVC than non-CVC patients (log-rank test, *P* < 0.001). Similarly, the rate of CV mortality was significantly higher among patients with CVC than non-CVC patients (log rank test, P<0.001).

A total of 434 MHD patients were enrolled, consisting of 62.2% men and 37.8% women. The mean age was 53.08 ± 13.39 years. The characteristics of the study population are listed in Table 1. CVC (47.2%) was observed in 434 patients: 118 (27.2%) had MVC, 138 (31.8%) had AVC, and 51 (11.8%) had both MVC and AVC. Compared to non-CVC patients, patients with CVC were older and had more comorbidities. They were less often users of vitamin D medications. Moreover, they had a higher LAD, IVST, and LVMI. Conversely, the serum albumin level was lower in patients with valve calcification than in those without CVC.

**Table 1:** Baseline clinical and lab characteristics in the CVC and non-CVC groups

	Non-CVC (229)	CVC (205)	*P*
Age, (years)	49.74 ± 13.87	56.81 ± 11.78	<0.001
Duration of HD, (years)	3.29 ± 3.33	3.58 ± 3.19	0.368
Men, n (%)	144 (62.88%)	126 (61.46%)	0.761
Smoking, n (%)	37 (16.16%)	26 (12.68%)	0.305
Hypertension history, n (%)	188 (82.10%)	172 (83.90%)	0.617
Diabetes, n (%)	44 (19.21%)	58 (28.29%)	0.026
Cardiovascular disease, n (%)	103 (44.98%)	137 (66.83%)	<0.001
Phosphorus-binding medications, n (%)	179 (78.17%)	147 (71.71%)	0.12
Vitamin D medications, n (%)	198 (86.46%)	159 (77.56%)	0.015
BMI (kg/m^2^)	22.98 ± 3.85	22.54 ± 3.72	0.179
LAD (cm)	3.38 ± 0.50	3.60 ± 0.55	<0.001
LVDd (cm)	4.74 ± 0.61	4.77 ± 0.73	0.94
IVST (cm)	1.19 ± 0.13	1.25 ± 0.17	<0.001
LVPWT (cm)	1.14 ± 0.13	1.15 ± 0.13	0.254
LVMI(g/m^2^)	119.38 ± 34.04	128.07 ± 38.10	0.013
EF	0.62 ± 0.08	0.61 ± 0.11	0.678
HGB (g/L)	105.15 ± 15.24	102.77 ± 16.82	0.122
Ca (mmol/L)	2.22 ± 0.23	2.24 ± 0.24	0.438
P (mmol/L)	2.09 ± 0.62	2.06 ± 0.62	0.558
β2-Microglobulin (mg/L)	29.29 ± 6.96	30.25 ± 7.25	0.158
iPTH (pg/mL)	389.75 ± 387.76	363.69 ± 382.43	0.379
ALB (g/L)	40.30 ± 3.37	39.34 ± 3.43	0.004
UA (μmol/L)	473.75 ± 96.25	460.57 ± 95.41	0.154
25(OH)D (ng/mL)	19.81 ± 10.76	19.45 ± 9.52	0.714

The univariate analyses and Cox proportional hazards models are shown in Table 2. In the univariable analysis, CVC, MVC, AVC, age, diabetes history, CVD history, use of phosphorus-binding medications, use of vitamin D medications, LAD, LVMI, EF, HGB, ALB, β2-microglobulin and iPTH were associated with all-cause mortality (Table 2). In the univariable analysis, CVC, MVC, AVC, age, diabetes history, CVD history, use of phosphorus-binding medications, use of vitamin D medications, LAD, LVMI, EF, HGB, ALB and β2-microglobulin were associated with CV mortality (Table 2).

**Table 2:** Univariate Cox analysis of factors in relation to all-cause and cardiovascular mortality

Variable	All-cause mortality		Cardiovascular mortality	
	HR(95%)	*P*	HR(95%)	*P*
CVC	2.647(1.955–3.583)	<0.001	5.992(3.877–9.261)	<0.001
MVC	1.927(1.434–2.589)	<0.001	3.161(2.251–4.439)	<0.001
AVC	2.650(1.988–3.533)	<0.001	4.177(2.951–5.912)	<0.001
Age	1.047(1.035–1.058)	<0.001	1.039(1.025–1.025)	<0.001
Duration of HD	0.971(0.928–1.017)	0.214	0.967(0.916–1.022)	0.231
Sex (male vs female)	0.922(0.687–1.238)	0.588	0.922(0.687–1.238)	0.893
Smoking	1.081(0.724–1.616)	0.703	1.267(0.808–1.985)	0.302
Hypertension history	1.162(0.782–1.727)	0.456	1.474(0.886–2.452)	0.135
Diabetes	2.797(2.078–3.765)	<0.001	2.984(2.106–4.228)	<0.001
CVD	1.713(1.268–2.315)	<0.001	2.571(1.748–3.780)	<0.001
Phosphorus-binding medications	0.492(0.354–0.683)	<0.001	0.588(0.392–0.883)	0.011
Vitamin D medications	0.427(0.317–0.576)	<0.001	0.437(0.306–0.622)	<0.001
BMI	1.003(0.966–1.043)	0.859	1.008(0.964–1.054)	0.734
LAD	1.817(1.419–2.328)	<0.001	2.292(1.729–3.037)	<0.001
LVMI	1.659(1.204–2.284)	0.002	1.890(1.281–2.790)	0.001
EF	0.197(0.043–0.912)	0.038	0.128(0.022–0.740)	0.022
HGB	0.984(0.975–0.993)	<0.001	0.982(0.972–0.993)	0.001
β2-Microglobulin	1.032(1.011–1.054)	0.003	1.026(1.001–1.052)	0.038
ALB	0.881(0.847–0.917)	<0.001	0.915(0.872–0.961)	<0.001
UA	0.999(0.997–1.000)	0.095	0.998(0.997–1.000)	0.099
25(OH)D	0.993(0.978–1.008)	0.338	0.992(0.974–1.009)	0.356
iPTH 150–300 pg/mL (as reference)				
iPTH < 150 pg/mL	1.516(1.033–2.226)	0.034	1.327(0.84–2.098)	0.225
iPTH > 300 pg/mL	1.081(0.741–1.575)	0.687	1.107(0.715–1.712)	0.687
Ca 2.1–2.5 mmol/L (as reference)				
Ca<2.1 mmol/L	1.091(0.792–1.504)	0.593	1.144(0.781–1.676)	0.488
Ca>2.5 mmol/L	0.954(0.603–1.508)	0.839	1.194(0.721–1.977)	0.490
P 1.13–1.78 mmol/L (as reference)				
P<1.13 mmol/L	1.24(0.593–2.591)	0.567	0.645(0.2–2.079)	0.463
P>1.78 mmol/L	0.897(0.659–1.222)	0.492	0.920(0.640–1.322)	0.653

Considering that MVC and AVC are the main manifestations of CVC in MHD patients, multivariate Cox proportional hazards analyses were conducted to identify factors associated with mortality. The presence of MVC was a significant factor associated with all-cause mortality (HR: 1.517, *P* = 0.010), in addition to old age, diabetes history, CVD history, no use of vitamin D medications, increased LAD, decreased HGB, increased β2-microglobulin and decreased ALB (Table 3). The presence of AVC was a significant factor associated with all-cause mortality (HR: 1.433, *P* = 0.028) in addition to old age, diabetes history, CVD history, no use of vitamin D medications, increased LAD, decreased HGB, increased β2-microglobulin and decreased ALB.

**Table 3:** Multivariate Cox regression models for all-cause mortality

Variable	MVC		AVC	
	HR(95%)	*P*	HR(95%)	*P*
mitral or aortic valve calcification	1.517(1.105–2.082)	0.010	1.433(1.039–1.976)	0.028
Age	1.028(1.015–1.041)	<0.001	1.026 (1.013–1.039)	<0.001
Diabetes	2.157(1.573–2.957)	<0.001	2.023(1.475–2.773)	<0.001
CVD	1.649(1.211–2.247)	0.002	1.625(1.186–2.225)	0.002
Phosphorus-binding medications	0.736(0.506–1.069)	0.107	0.773(0.528–1.132)	0.186
Vitamin D medications	0.736(0.506–1.069)	0.036	0.702(0.496–0.994)	0.046
LAD	1.506(1.067–2.125)	0.020	1.562(1.104–2.209)	0.012
LVMI	0.999(0.994–1.004)	0.616	0.998 (0.993–1.004)	0.569
EF	0.639(0.108–3.771)	0.621	0.657(0.107–4.017)	0.649
HGB	0.988(0.979–0.997)	0.011	0.988 (0.979–0.997)	0.011
β2-Microglobulin	1.022(1.000–1.045)	0.046	1.030 (1.008–1.053)	0.007
ALB	0.942(0.901–0.985)	0.009	0.947 (0.905–0.990)	0.015
iPTH 150–300 pg/mL (as reference)				
iPTH < 150 pg/mL	1.317(0.873–1.987)	0.189	1.240 (0.822–1.870)	0.304
iPTH > 300 pg/mL	1.371(0.925–2.032)	0.116	1.289 (0.877–1.912)	0.206

A Cox proportional hazards regression analysis of risk factors for CV mortality is shown in Table 4. After multivariate analysis, MVC (HR: 2.340, *P* < 0.001), diabetes history (HR: 2.538, *P* < 0.001), CVD history (HR: 2.284, *P* < 0.001), no use of vitamin D medications (HR: 0.579, *P* = 0.007), increased LAD (HR: 1.977, *P* = 0.001) and decreased HGB (HR: 0.987, *P* = 0.018) were associated with increased CV mortality. Moreover, AVC (HR: 2.410, *P* < 0.001), diabetes history (HR: 2.195, *P* < 0.001), CVD history (HR: 2.141, *P* < 0.001), no use of vitamin D medications (HR: 0.602, *P* = 0.013), increased LAD (HR: 2.157, *P* = 0.001) and decreased HGB (HR: 0.986, *P* = 0.016) were associated with increased CV mortality.

**Table 4:** Multivariate Cox regression models for cardiovascular mortality

Variable	MVC		AVC	
	HR(95%)	*P*	HR(95%)	*P*
mitral or aortic valve calcification	2.340 (1.625–3.370)	<0.001	2.410 (1.632–3.559)	<0.001
Age	1.015 (1.000–1.030)	0.056	1.010 (0.994–1.026)	0.223
Diabetes	2.538 (1.747–3.688)	<0.001	2.195 (1.507–3.197)	<0.001
CVD	2.284 (1.535–3.398)	<0.001	2.141 (1.443–3.179)	<0.001
Phosphorus-binding medications	0.847 (0.543–1.319)	0.462	0.952 (0.602–1.505)	0.832
Vitamin D medications	0.579 (0.389–0.864)	0.007	0.602 (0.403–0.899)	0.013
LAD	1.977 (1.353–2.889)	0.001	2.157 (1.455–3.197)	0.001
LVMI	0.997 (0.991–1.003)	0.282	0.996 (0.990–1.002)	0.207
EF	0.597 (0.080–4.438)	0.615	0.872 (0.108–7.031)	0.897
HGB	0.987 (0.976–0.998)	0.018	0.986 (0.975–0.997)	0.016
β2-Microglobulin	1.018 (0.994–1.044)	0.145	1.035 (1.010–1.061)	0.007
ALB	0.978 (0.928–1.032)	0.420	0.990 (0.940–1.042)	0.691

## Discussion

In the present study, nearly half of MHD patients had MVC and/or AVC. The presence of MVC and/or AVC was associated with poor survival in this population, and both MVC and AVC were independently associated with an increased risk of all-cause mortality and CV mortality.

CVC screening of dialysis patients is performed with various imaging techniques, such as plain X-ray radiography, computed tomography (CT) and echocardiography. The use of CT for the diagnosis of MVC and AVC is highly reliable and sensitive but has several disadvantages, such as radiation exposure and high cost [12]. The echocardiographic evaluation of CVC is an easy, reproducible, noninvasive, and inexpensive method and is recommended as a routine examination for CVC screening by the KDIGO guidelines [13].

Many studies have demonstrated the predictive value of valve calcification for mortality; however, some controversies remain. A cohort study in Japan suggested that the presence of CVC could predict cardiovascular and all-cause mortality in incident hemodialysis patients [3]. MVC and AVC were associated with a significantly increased risk for all-cause mortality in 144 hemodialysis patients, who were followed for a median of 5.6 years. However, after adjustment for multiple factors, MVC remained associated with all-cause mortality, whereas AVC was not [14]. A retrospective cohort study among 183 long-term hemodialysis patients showed that AVC increased the risk of cardiovascular death, whereas MVC did not [15]. Our study demonstrated that after adjusting for multiple factors, both MVC and AVC increased the risk of all-cause and CV mortality. The reasons for this finding may be the type of valve dysfunction that calcification causes. MVC may lead to mitral regurgitation, stenosis or both, whereas AVC can more often cause aortic stenosis. MVC has been directly related to an increased risk of CVD events, including coronary artery disease, acute myocardial infarction, stroke, and vascular diseases [16]. Aortic sclerosis was associated with a 50% higher risk of cardiovascular mortality and a 42% increased risk of myocardial infarction in the elderly [17]. Our findings may have important clinical implications. CVC on routine echocardiography should not be overlooked and can be used for risk assessment in MHD patients. Moreover, it can help identify patients who are appropriate for intensive medical treatment to reduce cardiovascular events.

In our study, we also focused on the relationship between the measured cardiac parameters and all-cause and cardiovascular death in MHD patients. LVMI was an independent risk factor in univariate Cox regression, but it was not significant in multivariate Cox regression after adjusting for other factors. However, LVMI is frequently reported as an independent mortality predictor among maintenance hemodialysis patients [18]. Accordingly, in other cohort studies, the progression of LVH was significantly related to all-cause mortality and cardiovascular events in 161 hemodialysis patients [19]. Our data show that LAD is significantly associated with all-cause and CV mortality, which is consistent with the results of previous studies. A study performed by Milan D et al. showed that a large left atrial diameter was an independent predictor of mortality in hemodialysis patients or renal allograft recipients [20, 21]. The left atrial dimensions greatly depend on the left ventricular pressure. When the ventricle is exposed to pressure and/or volume overload, such as in heart failure, hypertension or valve disease, this mechanism results in progressive left atrial enlargement. Left atrial dysfunction has been observed in association with mitral stenosis [22]. Left atrial pressure and volume overload provoke functional alterations in the atrial myocardium in mitral stenosis and correlate with exercise capacity in other causes of heart failure, which promote cardiac myocyte overstretching [23]. Mitral stenosis can cause left atrial enlargement from pressure or volume overload. A prospective study involving 249 patients with ESRD reported that dialysis patients who died during the follow-up had a higher LAD than survivors [24]. Thus, an increased LAD may signify increased cardiovascular risk in patients with CKD.

One of the other important findings of this study is that MHD patients who received oral active vitamin D treatment had significantly lower risks of cardiovascular and all-cause mortality. Experimental models suggest that activated vitamin D attenuates the development of LVH, improves left ventricular diastolic function, and reduces episodes of heart failure [25, 26]. In observational studies, MHD patients with a higher mortality risk may not have received vitamin D analogues, leading to an apparent association between vitamin D administration and better survival [27]. In this historical cohort study, chronic hemodialysis patients in the group that received injectable vitamin D showed a significant survival advantage over patients who did not [28]. However, the PRIMO and OPERA studies failed to demonstrate improvements in clinically relevant outcomes but demonstrated an increased risk of hypercalcaemia in CKD [29, 30]. The participants in the PRIMO and OPERA trials only had moderately increased PTH levels. In addition, active vitamin D will increase the intestinal calcium and phosphate absorption, which will worsen the vascular calcification. Thus, for severe and progressive secondary hyperparathyroidism (SHPT) in CKD G5D, calcitriol or vitamin D analogues should be considered, with an initially low dose, independent of the initial PTH concentration, and then titrated based on the PTH response, and hypercalcaemia should be avoided [6]. An observational study of chronic hemodialysis patients suggested a decrease in CV mortality among patients who received vitamin D to confirm the possibility that such medication improves the survival rate of ESRD patients [31]. We believe that our findings may have important clinical implications when vitamin D supplementation is considered a relatively easy, safe and inexpensive therapy in MHD patients suffering from SHPT. Further randomized and controlled studies are necessary to confirm the beneficial effect of vitamin D in lowering the cardiovascular mortality risk in patients with ESRD.

The major limitations of this study are its retrospective, single-centre design and a possible selection bias of excluding some patients without echocardiography or other examination data. Another limitation of this study is that cardiac ultrasound results were collected to evaluate whether there was valve calcification at the time of enrolment. However, the progression of CVC was not followed up. The strengths of our study include the relatively large number of participants, the long follow-up duration, and the ability to adjust for several established risk factors and potential confounders.

## Conclusions

In summary, our study demonstrated that CVC, including MVC and AVC, increases the risk of all-cause and cardiovascular mortality in MHD patients. Regular follow-up by echocardiography could be a useful method for risk stratification in MHD patients. Multicentre, large-sample, prospective cohort, follow-up studies are needed to confirm the reproducibility of our results and determine whether intervening in the progression of CVC can reduce the mortality rate among MHD patients.

## Data Availability

Data that support the results in the article can be found by academic researchers by sending an email to the corresponding author at szxyjh@aliyun.com.

## Abbreviations

CVC: Cardiac valve calcification
ESRD: End stage renal disease
CV: Cardiovascular
MVC: Mitral valve calcification
AVC: Aortic valve calcification
CVD: Cardiovascular disease
iPTH: Intact parathyroid hormone
25(OH)D: 25 hydroxy vitamin D
LV-EF: Left ventricular ejection fraction
LAD: Left atrial dimension
LVDd: Left ventricular end-diastolic internal dimension
LVPWT: Left ventricular posterior wall thickness
IVST: Interventricular septal wall thickness
LVMI: Left ventricular mass index
LVM: Left ventricular mass
